# Atg27p localization is clathrin- and Ent3p/5p-dependent

**DOI:** 10.17912/micropub.biology.000381

**Published:** 2021-03-29

**Authors:** Verónica A. Segarra, Anupam Sharma, Sandra K. Lemmon

**Affiliations:** 1 Department of Biology, High Point University, High Point, NC, USA 27268; 2 Department of Microbiology, University of Georgia, Athens, GA, USA 30602; 3 Department of Molecular and Cellular Pharmacology, University of Miami Miller School of Medicine, Miami, FL, USA 33101

## Abstract

The autophagy-related protein Atg27p has been previously shown to localize to the autophagy-specific pre-autophagosomal structure (PAS) as well as to several organelles, including the late Golgi, the vacuolar membrane, and the endosome. Given that Atg27p localization to the vacuolar membrane in particular has been shown to be dependent on both its C-terminal tyrosine sorting motif and the AP-3 adaptor, and that Atg27p can be found in clathrin-coated vesicles, we set out to determine whether Atg27p localization inside cells is dependent on clathrin or on any of its cargo adaptors. We report that Atg27p localization is clathrin- and Ent3p/5p-dependent.

**Figure 1. Atg27p displays clathrin- and Ent3p/5p-dependent localization in budding yeast:**
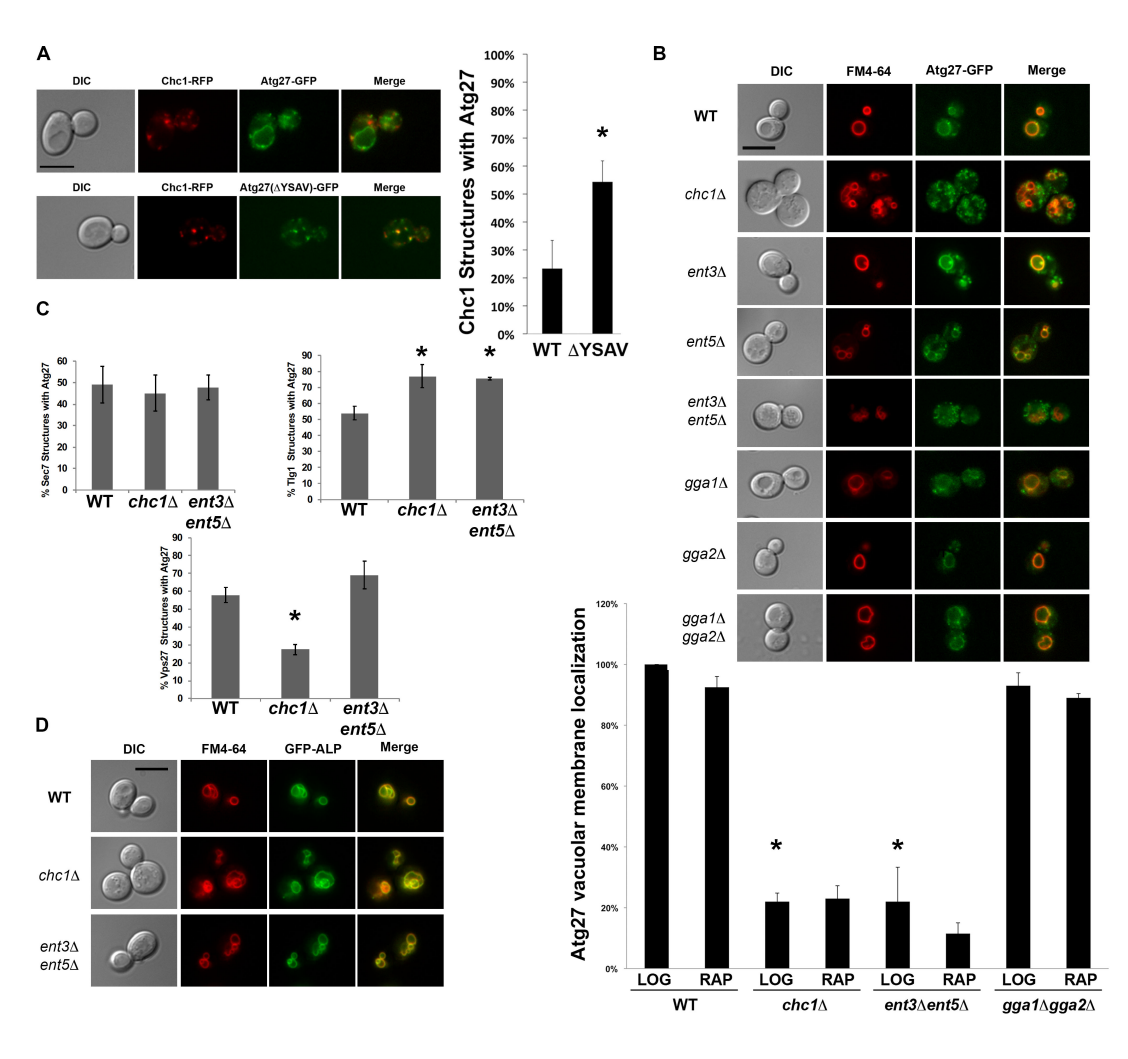
(A) Atg27p co-localizes with Chc1p with or without its C-terminal tyrosine sorting motif (YSAV). The percentage of Chc1p structures co-localizing with Atg27p (with or without its YSAV motif) was quantified (n ≥ 200, combined from three independent experiments). (B) Vacuolar membrane localization of Atg27p-GFP, shown previously to be an AP-3 cargo, is abrogated in cells deleted for *CHC1* and in the clathrin adaptor double mutant *ent3Δ ent5Δ*, but is still present in the *gga1Δ gga2Δ* clathrin adaptor double mutant with or without rapamycin*.* Individual deletion of the *ENT3*, *ENT5*, *GGA1*, or *GGA2* clathrin adaptor genes has no effect on Atg27p localization.Bar graphs indicate the percentage of FM4-64-stained vacuolar membranes containing Atg27p-GFP in the indicated mutant backgrounds (n ≥ 100, combined from three independent experiments) during logarithmic growth or after induction of autophagy by treatment with rapamycin. All micrographs presented were captured during logarithmic growth. Error bars indicate standard deviation (SD). *p* values less than 0.05, relative to the wildtype/logarithmic growth conditions are indicated by an asterik (*). (C) Quantification of co-localization of Atg27p-GFP with Golgi (Sec7p) and endosomal (Tlg1p early; Vps27p late) markers in clathrin and *ent3*/*ent5* mutants. Bar graphs indicate the percentage of indicated Golgi/endosomal structures containing Atg27p‐GFP (n ≥ 100, combined from two independent experiments for the wild type strain and three independent experiments for all other strains). Error bars indicate standard deviation (SD). *p* values less than 0.05, relative to the wildtype/logarithmic growth conditions are indicated by an asterik (*). (D) Classical AP-3 cargo alkaline phosphatase (ALP) displays traditional clathrin-independent, as well as *ent3∆ ent5∆*-independent localization to the vacuolar membrane. Scale bars = 5 microns.

## Description

The autophagy-related protein Atg27p has been previously shown to localize to the autophagy-specific pre-autophagosomal structure (PAS) as well as to several organelles, including the late Golgi or trans-Golgi network (TGN), the vacuolar membrane, and the early and late endosomes (Segarra *et al.* 2015). Moreover, Atg27p localization to the vacuolar membrane, in particular, is dependent on both its C-terminal tyrosine sorting motif and the AP-3 adaptor (Segarra *et al.* 2015; Suzuki and Emr 2019), but not other yeast AP-related adaptors (Segarra *et al.* 2015). We recently reported that Atg27p can be found in clathrin-coated vesicles (CCVs; Ding, Segarra *et al.* 2016, See accompanying micropublication). For this reason, we set out to determine whether Atg27p localization is dependent on clathrin or on any other of its cargo adaptors.

We found that Atg27p co-localizes with Chc1p (Figure 1A), even in the absence of its C-terminal tyrosine sorting motif YSAV (ΔYSAV). Thus, the presence of Atg27p in CCVs does not seem to require the anterograde transport of Atg27p to the vacuolar membrane. Atg27p mutant molecules deleted for their YSAV motif display increased colocalization with clathrin (Figure 1A). AP-3 sorting to the vacuole of these ΔYSAV mutants is most likely impaired, leading to increased amounts of Atg27p entering the traditional TGN/endosomal pathway.

To examine the overall localization pattern of Atg27p in cells lacking clathrin, we imaged cells expressing GFP-tagged Atg27p that were deleted for *CHC1*. In *chc1∆*, Atg27p localizes to small punctate structures throughout the cell and its vacuolar membrane localization is lost (Figure 1B). This was surprising because Atg27p has been shown to be an AP-3 cargo (Segarra *et al.* 2015) and the trafficking of AP-3 cargo, such as alkaline phosphatase (ALP; Stepp *et al.* 1997; Cowles *et al.* 1997), has traditionally been considered to be clathrin-independent (Vowels and Payne 1998). Similarly, we examined vacuolar membrane localization of Atg27p in cells missing the other TGN/endosomal clathrin adaptors, epsins or ggas. Double deletion of the *ENT3* and *ENT5* adaptor genes appear to recapitulate the vacuolar membrane localization defect seen in the clathrin-null cells, suggesting that transport of Atg27p is also dependent on the Ent3p and Ent5p adaptor proteins (Figure 1B). Ent5p was also identified in the mass spectrometry screen for CCV components, similar to Atg27p (WT screen, Ding, Segarra *et al.* 2016). The Ent3p and Ent5p epsin-like proteins have been shown previously to have separate roles in clathrin-mediated TGN/endosomal traffic (Costaguta, Duncan *et al.* 2006). Our finding that the Atg27p localization phenotype in clathrin null cells can only be recapitulated by combined deletion of *ENT3* and *ENT5* may indicate that clathrin is acting at more than one step in Atg27p TGN/endosomal transport. It is also interesting that the vacuolar membrane localization of Atg27p is not affected in any of the GGA adaptor mutants, as GGA clathrin adaptors were shown by Casler and Glick to be required for traffic of vacuolar cargo from the maturing Golgi (2020).

To determine the cellular location of the small Atg27p puncta in the *chc1∆* and *ent3∆ ent5∆* mutants, we quantified their co-localization with traditional Golgi/endosomal markers Sec7p, Tlg1p, and Vps27p. Atg27p puncta in the clathrin-null and the *ent3∆ ent5∆* double deletion mutants partially localize to the Sec7p- and Tlg1p-marked TGN/recycling endosome and the Vps27p-marked pre-vacuolar endosome (Figure 1C). The budding yeast TGN has been shown to serve as the cell’s early/recycling endosome (Day *et al.* 2018), potentially explaining why certain organellar markers such as Sec7p and Tlg1p are sometimes reported to co-localize (Grissom *et al.* 2020). All in all, we can conclude that the Atg27p puncta in the clathrin null and in the *ent3∆ ent5∆* cells partially localize to TGN/recycling endosome and the pre-vacuolar endosome.

The observed loss of vacuolar membrane localization was specific to Atg27p and not to all AP-3 cargoes, since ALP was able to localize to the vacuolar membrane in the clathrin and *ent3* *ent5* mutants (Figure 1D). This suggests that Atg27p is a non-canonical AP-3 cargo in that its vacuolar membrane localization is both AP-3 and clathrin- and epsin-dependent. Recent studies showed that Atg27p is recycled from the vacuolar membrane to endosomes and then the TGN (Suzuki and Emr 2018). Possibly, in the absence of clathrin or the epsins, Atg27p recycling to the TGN is impaired, leading to the loss of Atg27p from the vacuolar membrane and accumulation in TGN/endosomal compartments. Of interest, in *chc1∆* cells, Atg27p was decreased on Vps27p-positive structures (Figure 1C), suggesting that clathrin’s major role is in a later retrieval step from the TGN.

## Methods

**Yeast and plasmid methods**

Standard methods and media were used for genetic manipulations, growth, and transformation of yeast (Guthrie and Fink 1991). To induce autophagy, log phase cells were treated with rapamycin (LC Laboratories, R‐5000) at 0.2 µg/mL for at least 2 hours at 30°C. *Saccharomyces cerevisiae* strains used in this study are listed in the table below. Unless otherwise indicated, the Longtine method was used for yeast construction (Longtine *et al.* 1998). pGFP-ALP was used as an ALP localization reporter (Cowles *et al.* 1997).

**Yeast strains used in this study**

**Table:** 

**Name**	**Alias**	**Genotype**	**Panel**	**Reference**
SL5970	Chc1-RFPAtg27-GFP	*MATa leu2 ura3 trp1 his3 lys2 CHC1-RFP::KANMX6 ATG27-GFP::HISMX*	1A	This Paper
SL6154	Chc1-RFP Atg27(ΔYSAV)-GFP	*MATa leu2 ura3 trp1 his3 lys2 CHC1-RFP::KANMX6 ATG27(-YSAV)-GFP::HISMX*		
SL5837	Atg27-GFP	*MAT*α *leu2 ura3-52 trp1 his3-∆200 ATG27-GFP::HISMX6*	1B	Segarra *et al.* 2015
SL6240	*chc1Δ* Atg27-GFP	*MATa leu2 ura3-52 trp1 his3-∆200 chc1∆::LEU2 ATG27-GFP::HISMX*		This paper
SL5945	*ent3Δ* Atg27-GFP	*MATa leu2 ura3-52 trp1 his3-∆200 ent3Δ::TRP1 ATG27-GFP::HISMX6*		
SL5946	*ent5Δ* Atg27-GFP	*MATa leu2 ura3-52 trp1 his3-∆200 ent5Δ::TRP1 ATG27-GFP::HISMX6*		
SL5947	*ent3Δ ent5Δ* Atg27-GFP	*MAT*α *leu2 ura3-52 trp1 his3-∆200 ent3Δ::TRP1 ent5Δ::TRP1 ATG27-GFP::HISMX6*		
SL6214	*gga1Δ* Atg27-GFP	*MAT*α *leu2 ura3-52 trp his3-∆200 lys2-801 gga1Δ::HIS3 ATG27-GFP::HISMX*		
SL6215	*gga2Δ* Atg27-GFP	*MAT*α *leu2 ura3-52 trp his3-∆200 lys2-801 gga2Δ::TRP1 ATG27-GFP::HISMX*		
SL6222	*gga1Δ gga2Δ* Atg27-GFP	*MAT*α *leu2 ura3-52 trp his3-∆200 lys2-801 gga1Δ::HIS3 gga2Δ::TRP1 ATG27-GFP::HISMX*		
SL7575	*chc1Δ* Atg27-GFP Sec7-dsRed	*MATa leu2 ura3-52 trp1 his3-∆200 scd1-v chc1∆::LEU2 ATG27-GFP::HISMX6 pSEC7-dsRed (316)*	1C	This paper
SL7576	*chc1Δ* Atg27-GFP mcherry-Tlg1	*MATa leu2 ura3-52 trp1 his3-∆200 scd1-v chc1∆::LEU2 ATG27-GFP::HISMX6 pRS316-mcherry-TLG1*		
SL7577	*chc1Δ* Atg27-GFP Vps27-RFP	*MATa leu2 ura3-52 trp1 his3- ∆ 200 scd1-v chc1∆::LEU2 ATG27-GFP::HISMX6 pRS316-VPS27-RFP*		
SL7572	*ent3∆ ent5∆* Atg27-GFP Sec7-dsRed	*MATα ura3-52 his3 ∆ -200 trp1901 leu2-3,112 lys2-801 suc2- ∆9 ent3∆::TRP1 ent5∆::TRP1 ATG27-GFP::HISMX6 pSEC7-dsRed (316)*		
SL7573	*ent3∆ ent5∆* Atg27GFP mcherry-TLG1	*MATα ura3-52 his3 ∆ -200 trp1901 leu2-3,112 lys2-801 suc2- ∆9 ent3∆::TRP1 ent5∆::TRP1 ATG27-GFP::HISMX6 pRS316-mcherry-TLG1*	1C	This paper
SL7574	*ent3∆ ent5∆* Atg27GFP Vps27-RFP	*MATα ura3-52 his3 ∆ -200 trp1901 leu2-3,112 lys2-801 suc2- ∆9 ent3∆::TRP1 ent5∆::TRP1 ATG27-GFP::HISMX6 pRS316-VPS27-RFP*		
SL7575	*chc1Δ* Atg27-GFP Sec7-dsRed	*MATa leu2 ura3-52 trp1 his3- ∆200 scd1-v chc1∆::LEU2 ATG27-GFP::HISMX6 pSEC7-dsRed (316)*		
SL7576	*chc1Δ* Atg27-GFP mcherry-Tlg1	*MATa leu2 ura3-52 trp1 his3- ∆ 200 scd1-v GAL2? chc1∆::LEU2 ATG27-GFP::HISMX6 pRS316-mcherry-TLG1*		
SL7577	*chc1Δ* Atg27-GFP Vps27-RFP	*MATa leu2 ura3-52 trp1 his3- ∆ 200 scd1-v GAL2? chc1∆::LEU2 ATG27-GFP::HISMX6 pRS316-VPS27-RFP*		
SL6679	*pGFP-ALP (SL6610)*	*MAT*α *leu2 ura3-52 trp1 his3-∆200* pGFP-ALP	1D	This paper
SL6681	*chc1Δ* GFP-ALP	*MAT*α *leu2 ura3-52 trp1 his3-∆200 chc1∆::LEU2* pGFP-ALP		
SL6680	*ent3Δ ent5Δ* GFP-ALP	*MAT*α *leu2 ura3-52 trp1 his3-∆200 ent3Δ::TRP1 ent5Δ::TRP1 pGFP-ALP*		

**Microscopy methods**

Vacuolar membrane staining with FM4-64 was carried out as described previously (Segarra *et al.* 2015). Microscopy was performed on an Olympus fluorescence BX61 upright microscope equipped with Nomarski differential interference contrast (DIC) optics, a Uplan S Apo 100x objective (NA 1.4), a Roper Cool-Snap HQ camera, and Sutter Lambda 10–2 excitation and emission filter wheels, and a 175 watt Xenon remote sourcewith liquid light guide. Image capture was automated using Intelligent Imaging Innovations Slidebook 4.01 for Mac. A series of optical Z-sections (0.25 μm) was captured for each cell analyzed. Prior to analysis, the stacks were deconvolved using the nearest neighbor algorithm. Representative single-plane micrographs from cells at log phase were chosen to be included in the figures.

To quantify co-localization of Atg27p with Chc1p or organellar markers, deconvolved Z-stacks were examined to confirm that both fluorescent signals were in the same plane, and that peak fluorescence overlapped in corresponding sections. Co-localization was expressed as the percent of structures of interest that contained the GFP-tagged Atg27p construct. To determine statistical significance, two-tailed Student’s *t*-tests were performed to compare each condition of interest to the WT control.
